# MAB21L1 loss of function causes a syndromic neurodevelopmental disorder with distinctive *c*erebellar, *o*cular, cranio*f*acial and *g*enital features (COFG syndrome)

**DOI:** 10.1136/jmedgenet-2018-105623

**Published:** 2018-11-28

**Authors:** Abolfazl Rad, Umut Altunoglu, Rebecca Miller, Reza Maroofian, Kiely N James, Ahmet Okay Çağlayan, Maryam Najafi, Valentina Stanley, Rose-Mary Boustany, Gözde Yeşil, Afsaneh Sahebzamani, Gülhan Ercan-Sencicek, Kolsoum Saeidi, Kaman Wu, Peter Bauer, Zeineb Bakey, Joseph G Gleeson, Natalie Hauser, Murat Gunel, Hulya Kayserili, Miriam Schmidts

**Affiliations:** 1 Genome Research Division, Human Genetics Department, Radboud University Medical Center, Nijmegen, The Netherlands; 2 Cellular and Molecular Research Center, Sabzevar University of Medical Sciences, Sabzevar, Iran; 3 Medical Genetics Department, İstanbul Medical Faculty, İstanbul University, Istanbul, Turkey; 4 Inova Cardiovascular Genomics Clinic, Inova Translational Medicine Institute, Falls Church, Virginia, USA; 5 Genetics and Molecular Cell Sciences Research Centre, St George’s, University of London, London, UK; 6 Laboratory for Pediatric Brain Disease, Howard Hughes Medical Institute, Rady Children’s Institute for Genomic Medicine, University of California, San Diego, California, USA; 7 Department of Neurosurgery, Program on Neurogenetics, Yale School of Medicine, Yale University, New Haven, Connecticut, USA; 8 Medical Genetics Department, Bilim University School of Medicine, İstanbul, Turkey; 9 Department of Pediatrics and Adolescent Medicine, Neurogenetics Program and Division of Pediatric Neurology, American University of Beirut Medical Center Special Kids Clinic, Beirut, Lebanon; 10 Biochemistry and Molecular Genetics, American University of Beirut, Beirut, Lebanon; 11 Medical Genetics Department, Bezmi Alem University School of Medicine, Istanbul, Turkey; 12 Paediatric and Genetic Counselling Center, Kerman Welfare Organization, Kerman, Iran; 13 Neuroscience Research Center, Institute of Neuropharmacology, Kerman University of Medical Sciences, Kerman, Iran; 14 Department of Medical Genetics, Kerman University of Medical Sciences, Kerman, Iran; 15 Centogene AG, Rostock, Germany; 16 Pediatrics Genetics Division, Center for Pediatrics and Adolescent Medicine, Faculty of Medicine, Freiburg University, Freiburg, Germany; 17 Medical Genetics Department, Koç University School of Medicine (KUSoM), İstanbul, Turkey

**Keywords:** *MAB21L1*, corneal dystrophy, scrotal/labial aplasia, pontocerebellar hypoplasia, Cerebello-Oculo-Facio-genital (COFG) syndrome

## Abstract

**Background:**

Putative nucleotidyltransferase MAB21L1 is a member of an evolutionarily well-conserved family of the male abnormal 21 (MAB21)-like proteins. Little is known about the biochemical function of the protein; however, prior studies have shown essential roles for several aspects of embryonic development including the eye, midbrain, neural tube and reproductive organs.

**Objective:**

A homozygous truncating variant in *MAB21L1* has recently been described in a male affected by intellectual disability, scrotal agenesis, ophthalmological anomalies, cerebellar hypoplasia and facial dysmorphism. We employed a combination of exome sequencing and homozygosity mapping to identify the underlying genetic cause in subjects with similar phenotypic features descending from five unrelated consanguineous families.

**Results:**

We identified four homozygous *MAB21L1* loss of function variants (p.Glu281fs*20, p.Arg287Glufs*14 p.Tyr280* and p.Ser93Serfs*48) and one missense variant (p.Gln233Pro) in 10 affected individuals from 5 consanguineous families with a distinctive autosomal recessive neurodevelopmental syndrome. Cardinal features of this syndrome include a characteristic facial gestalt, corneal dystrophy, hairy nipples, underdeveloped labioscrotal folds and scrotum/scrotal agenesis as well as cerebellar hypoplasia with ataxia and variable microcephaly.

**Conclusion:**

This report defines an ultrarare but clinically recognisable Cerebello-Oculo-Facio-Genital syndrome associated with recessive *MAB21L1* variants. Additionally, our findings further support the critical role of MAB21L1 in cerebellum, lens, genitalia and as craniofacial morphogenesis.

## Introduction

*MAB21l1* belongs to the conserved male abnormal gene family 21 (mab21). First described in the nematode *Caenorhabditis elegans* as a transcription factor in cell fate determination,^1^ the family consists of three members, namely *mab21l1*, *mab21l2* and *mab21l3* in vertebrates. Mab21 genes play a major role in embryonic development but gene expression extends beyond the developmental period well into adulthood.^2^ Molecular explorations in *C. elegans*, *Danio rerio*, *Xenopus tropicalis* and mice indicate a crucial role for Mab21 family members in diverse, developmentally important cell signalling pathways, including TGF-B/BMP, JNK1/MKK4, PAX6. Members of these pathways have been previously shown to play an important role for lens development and their expression patterns correlate with different *Hox* genes.^3–5^

Protein modelling of Mab21L1 and Mab21L2 in vertebrates shows 94% identical amino acid sequences, raising the possibility of partially redundant gene function.^6^ Likewise, Mab21l1 and Mab21l2 expression patterns in vertebrates are partially overlapping in the developing eye, midbrain, branchial arches and limb buds.^3^ Both Mab21l1 and Mab21l2 depleted mouse embryos show severe defects in development of the notochord, neural tube and organogenesis, vasculogenesis and axial turning.^7^ Intriguingly, *Mab21l1*-/- mice have defects only in ocular development and preputial glands and very recently demonstrated an unclosed calvarium.^4 8^ Hypomorphic *mab21 C. elegans* mutants develop a short and fat body, uncoordinated movement and reduced fertility in addition to defects in sensory rays.^9^ In humans, recessive *MAB21L2* alleles have been associated with a range ocular malformations such as microphthalmia/anophthalmia, coloboma, with skeletal dysplasias and intellectual disability (ID).^10 11^

Recently, a truncating *MAB21L1* variant (c.735dupG; p.Cys246Leufs*18) was identified in a male affected with ID, scrotal agenesis, ophthalmological anomalies, cerebellar malformation and facial dysmorphism.^12^ Of note, this extremely rare combination of clinical findings was previously reported in four patients from two families in the literature.^13^ Comparison of phenotypic data between the reported cases and the *MAB21L1* index case may suggest a common syndrome; however, no molecular investigations were reported in those families.

Here, we now report novel biallelic variants in 10 patients from five independent families with Cerebello-Oculo-Facio-Genital (COFG) syndrome, confirming *MAB21L1* loss of function is the underlying cause for this ultrarare hereditary disorder.

## Material and methods

### Subjects

Recruitment criteria to the study were syndromic labioscrotal aplasia and/or cerebellar hypoplasia with eye/genital anomalies. DNA samples were collected after obtaining informed patient or parental consent as part of the clinical diagnostic pathway at Radboud University Medical Center Nijmegen (Innovative Diagnostic Programme), as part of the clinical genetics diagnostic pathway at Inova Fairfax Hospital, Falls Church, Virginia, USA, Laboratory for Paediatric Brain Disease, Howard Hughes Medical Institute, Rady Children’s Institute for Genomic Medicine, University of California, San Diego, California, USA, Department of Neurosurgery, Program on Neurogenetics, Yale School of Medicine, Yale University, New Haven, Connecticut, USA and Istanbul Koc University or as part of a research study with the approval of the Institutional Ethics Review Board (approval number KUSoM; 2015.120.IRB2.047 CRANIRARE-2, HIC # 94060007680).

### Exome sequencing (ES)

Exomic sequences from DNA samples of family 1 were enriched with the SureSelect Human All Exon 50 Mb V.6 Kit (Agilent Technologies, Santa Clara, California, USA) Kit (families 1, 4 and 5) and exonic regions and flanking splice junctions of the genome were captured using a proprietary system developed by GeneDx (Gaithersburg, Maryland, USA) for family 2. For family 3, Nextera Rapid Capture Exome Kit (Illumina, San Diego, California, USA) was used for exome capture. 100 bp paired-end reads were generated either on Hiseq PE150 (Illumina) or using a HiSeq2000 or HiSeq4000 with paired end analysis. Image analysis and subsequent base calling was performed using the Illumina pipeline (V.1.8). Read alignment and variant calling were performed with DNAnexus (Palo Alto, California, USA) using default parameters with the human genome assembly hg19 (GRCh37) as reference for families 1, 2 and 4. For family 3, a Centogene in-house pipeline previously described was used for variant calling instead^14^ while likewise for family 5, we used a different in-house written variant calling pipeline as described previously.^15^ Briefly, sequence reads were mapped to the human genome reference sequence (version GRCh37, as used in phase 1 of the 1000 Genomes Project) using a hybrid of Stampy^16^ and BWA.^17^ For all five families, variant calling of SNVs and small indels was accomplished using the Unified Genotyper algorithm from GATK.^18^ Variant alleles were annotated using Ensembl database (V.66) and Variant Effect Predictor (V.2.4) tool.^19^ Following alignment and variant calling, serial variant filtering was performed for variants a MAF equal or less than 0.5% for families 1–3 and less than 0.1% for families 4 and 5 inExAc, 1000 genome project, esp6500 databases and gnomad. For family 5, an additional in-house exome database for Turkish exomes was used. Coding variants include frameshift, stop and variants changing the protein structure using scores from SIFT (Sorting Intolerant From Tolerant; http://sift.bii.a-star.edu.sg), PolyPhen (http://genetics.bwh.harvard.edu/pph2) and GERP++ (Genomic Evolutionary Rate Profiling; http://mendel.stanford.edu/SidowLab/downloads/gerp) or variants within 5 bp of exon-intron boundaries and genes carrying biallelic variants with prioritisation of homozygous variants in consanguineous pedigrees and genes with compound variants in non-consanguineous pedigrees. Obligate loss of function variants such as canonical splice variants, frameshift and stop mutations were prioritised over missense variants; however, missense variants were not excluded from the analysis. For family 2, variants were filtered using a custom-developed analysis tool (XomeAnalyzer), data were filtered and analysed to identify sequence variants and most deletions and duplications involving three or more coding exons (GeneDx).

### Sanger sequencing

Genomic DNA was isolated by standard methods directly from blood samples using a standard DNA extraction kit (Qiagen, USA). Amplification of genomic DNA was performed in a volume of 50 µl containing 30 ng DNA, 50 pmol of each primer, 2 mM dNTPs and 1.0 U GoTaq DNA polymerase (Promega Corporation, #M3001) or 1.0 U MolTaq polymerase (Molzym Corporation, #P-016). PCR amplifications were carried out by an initial denaturation step at 94°C for 3 min and 33 cycles as follows: 94°C for 30 s, 58–60°C for 30 s and 72°C for 70 s, with a final extension at 72°C for 10 min. PCR products were verified by agarose gel electrophoresis, purified and sequenced bidirectionally. The sequence data were evaluated using the CodonCode or Sequencher 4.9 (Gene Codes) software. Primer sequences are available on request.

### Genomic SNP array

Genome-wide single nucleotide varaint (SNP) array analysis was undertaken in families 1 and 2 using either Illumina Global Screening Array (Neuherberg, Germany) (family 1) or postnatal, Oligo-SNP array Quest Diagnostics (Chantilly, Virginia, USA) (family 2) according to the manufacturer’s protocol (http://www.illumina.com/techniques/microarrays).

### String protein-protein interaction network analysis

We used the string protein interaction network online tool at https://string-db.org/ 20 to search for Mab21l1 binding partners. We chose an analysis setup where only experimentally determined interaction partners are displayed. Confidence level for all interaction proteins was high as defined by String (>0.70).

## Results

**Figure 1:**
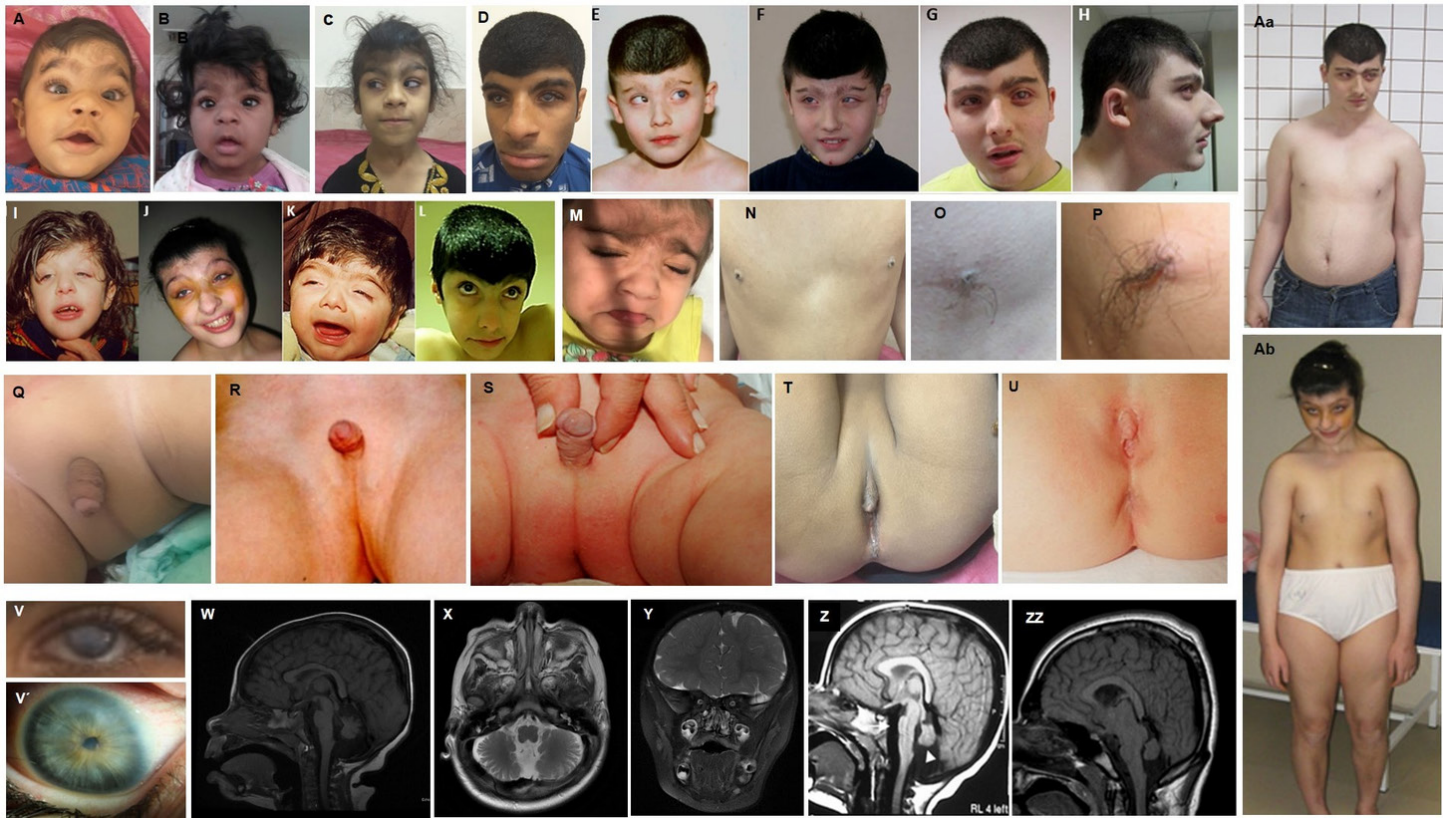
Clinical phenotype of COFG individuals harbouring biallelic MAB21L1 loss of function variants. (A–L) Depicted craniofacial dysmorphism, medially sparse/flared and laterally extending eyebrows, synophrys, buphthalmos, anteverted nares, long and tented philtrum, flat nasal bridge, low anterior hairline and horizontal nystagmus with strabismus (A–D: individuals from family 1; E–H and Aa: individual 4_V:1 from 7 years of age to adulthood; I, J, Ab: individual 5_IV:1 from childhood to adulthood; K, L: individual 4_IV.4 from childhood to adulthood, M: individual 3_II.3). Widely spaced hypoplastic nipples with no noticeable areolae or hyperpigmentation and a tuft of terminal hair extruding from the lactiferous ducts (N: individual 1_VI:7; O: individual 4_V:1; P: 5_IV:4). Undescended testes, bilateral scrotal agenesis with normal median raphe in individual 1_VI:2 (Q), 4_V:1 (R), 5_IV:4 (S). Absence of labia majora and small labia minora in individuals 1_VI:5 (T) and 5_IV:1 (U). Corneal opacities in individual 1_VI:7 (V) and individual 5_IV:1. (V`). Cerebellar hypoplasia with absence/hypoplasia of the vermis in individuals 2_II:3 (W, X), 4_V:1 (Z, I_IV.V). Optic atrophy in individual 2_II:3 (Y). COFG, Cerebello-Oculo-Facio-Genital.

In order to elucidate the genetic cause of the strikingly similar syndromic labioscrotal aplasia phenotype in four consanguineous families, we performed exome sequencing (ES). Clinical features are displayed in figure 1 and summarised in table 1.

**Table 1:** Clinical findings in individuals harbouring biallelic MAB21L1 variants

	Family 1				Family 2	Family 3	Family 4*	Family 5*			Family 6†
	Patient 1	Patient 2	Patient 3	Patient 4	Patent 5	Patient 6	Patient 7	Patient 8	Patient 9	Patient 10	Patient 11
MAB21L1 mutation	c.841delG, p.Glut281fs*20				c.698 A>C, p.Gln233Pro	c.279_286delACTGCCCG (p.Ser93Serfs*48)	c.859delC, p.Arg287Glufs*14	c.840C>G; p.Tyr280*			c.735dupG, p.Cys246Leufs*18
Ancestry	Persian				Persian	Lebanese Shia	Turkish	Turkish			Algerian
Sex	Female	Female	Male	Male	Male	Female	Male	Female	Male	Female	Male
Consanguinity	1° cousins				1° cousins	1° cousins	2° cousins	1° cousins			1° cousins
Growth parameters											
Measurements at birth (SD)	3.5 kg (0.81), n/a, 33 cm (−1.22)	3.1 kg (−0.62), n/a, 34 cm (−0.54)	3.1 kg (−0.74), 49 cm (−0.44), n/a	n/a	3.6 kg (+0.08), 56 cm (+2.23), 34 cm (−0.54)	2.8 kg (−1.19), 49 cm (−0.20), 35 cm (+0.11)	3.45 kg (−0.18), 50 cm (−0.06), 34 cm (+0.54)	n/a	3.5 kg (−0.10), n/a, n/a	n/a	n/a, n/a, n/a
Age at last evaluation	7 years	5 years	7 months	17 years	26 months	2 years	18 years 9 months	16 years	10 years 6 months	12 months	7 years
Height (SD)	119 cm (−0.47)	106 cm (−0.36)	67 cm (−0.62)	157 cm (2.10)	n/a	87.5 cm (+0.36)	170 cm (−0.92)	165 cm (+0.43)	130 cm (−1.57)	n/a	116 cm (−1.7)
Weight (SD)	18 kg (−1.75)	14 kg (−2.02)	7 kg (−1.57)	47 kg (−1.91)	n/a	11.1 kg (−0.96)	74 kg (+0.31)	50 kg (−0.50)	38 kg (+0.48)	n/a	20 kg (−1.18)
OFC (SD)	49 cm (−1.96)	52 cm (+1.19)	41 cm (−2.47)	55.5 cm (−0.07)	n/a	47.5 cm (+0.02)	52 cm (−2.09)	46 cm (−6.86)	45.8 cm (−5.52)	41.5 cm (−3.20)	50 cm (−1.50)
Craniofacial findings											
Short forehead	+	+	+	+	+	+	+	+	+	+	+
Medially sparse/flared and laterally extended eyebrows	+	+	+	+	+	+-	+	+	+	+	+
Low-set ears	–	–	–	–	–		+	+	–	n/a	+
Ocular abnormalities											
Horizontal nystagmus	+	+	+	in infancy	+	–	+	+	+	+	+
Bilateral corneal opacities/corneal dystrophy	+	+	+	+	+	+	+	+	+	+	+
Genitourinary findings/breasts											
Absent scrotum/labia majora	+	+	+	+	+	+	+	+	+	+	+
Hypospadias	NA	NA	–	–	+	NA	-	NA	+	NA	-
Tuft of hair extruding from the lactiferous ducts	–	–	–	+	n/a	n/a	+	+	+	+	+‡¥
Neurological features											
Global DD/ID	Severe	Severe	n/a	Moderate	n/a	Severe	Moderate to severe	Moderate	Moderate	Severe	Severe
Cranial MRI findings	Cerebellar hypoplasia, Dandy-Walker malformation	n/a	n/a	Cerebellar hypoplasia, Dandy-Walker malformation	Cerebellovermian hypoplasia, optic nerve hypoplasia	Cerebellovermian hypoplasia with pontine involvement	Severe cerebellar and vermian hypoplasia and large parietal cyst	Cerebellovermian hypoplasia with pontine involvement	Cerebellovermian hypoplasia with pontine involvement	Cerebellovermian hypoplasia with pontine involvement	Dandy-Walker malformation
Endocrinological evaluation											
Basal testosterone	NA	NA	n/a	n/a	Normal	NA	3.20 ng/mL (2.7–10.7)	NA	0.02 ng/mL (0.07–0.2)	NA	Normal
Basal estradiol	n/a	n/a	NA	NA	NA	n/a	NA	68.55 pg/mL (0–266)	NA	n/a	NA
Response to short stimulation test	NA	NA	n/a	n/a	n/a	NA	Adequate	NA	Adequate	NA	n/a
LH, FSH	n/a	n/a	n/a	n/a	n/a	n/a	4.6 mIU/mL (1.7–8.6) 5.4 mIU/mL (1.5–12.4)	6.13 mIU/mL (1.6–8.3) 3.81 mIU/mL (3–10)	0.03 mIU/mL (0.3–2.8) 0.68 mIU/mL (<4.1)	n/a	Normal
Other											
	–	–	–	Heart murmur	Retinal degeneration	Decreased sensitivity to pain	–	–		–	–

*Patients’ phenotypes previously published.^13^

†Patient phenotype and causative *MAB21L1* mutation previously published.^12^

‡Personal communication with J. Thevenon.

DD, developmental delay; FSH, follicle stimulating hormone; ID, intellectual disability; LH, luteinising hormone; n/a, not available; NA, not applicable; +, present; –, absent.

**Figure 2:**
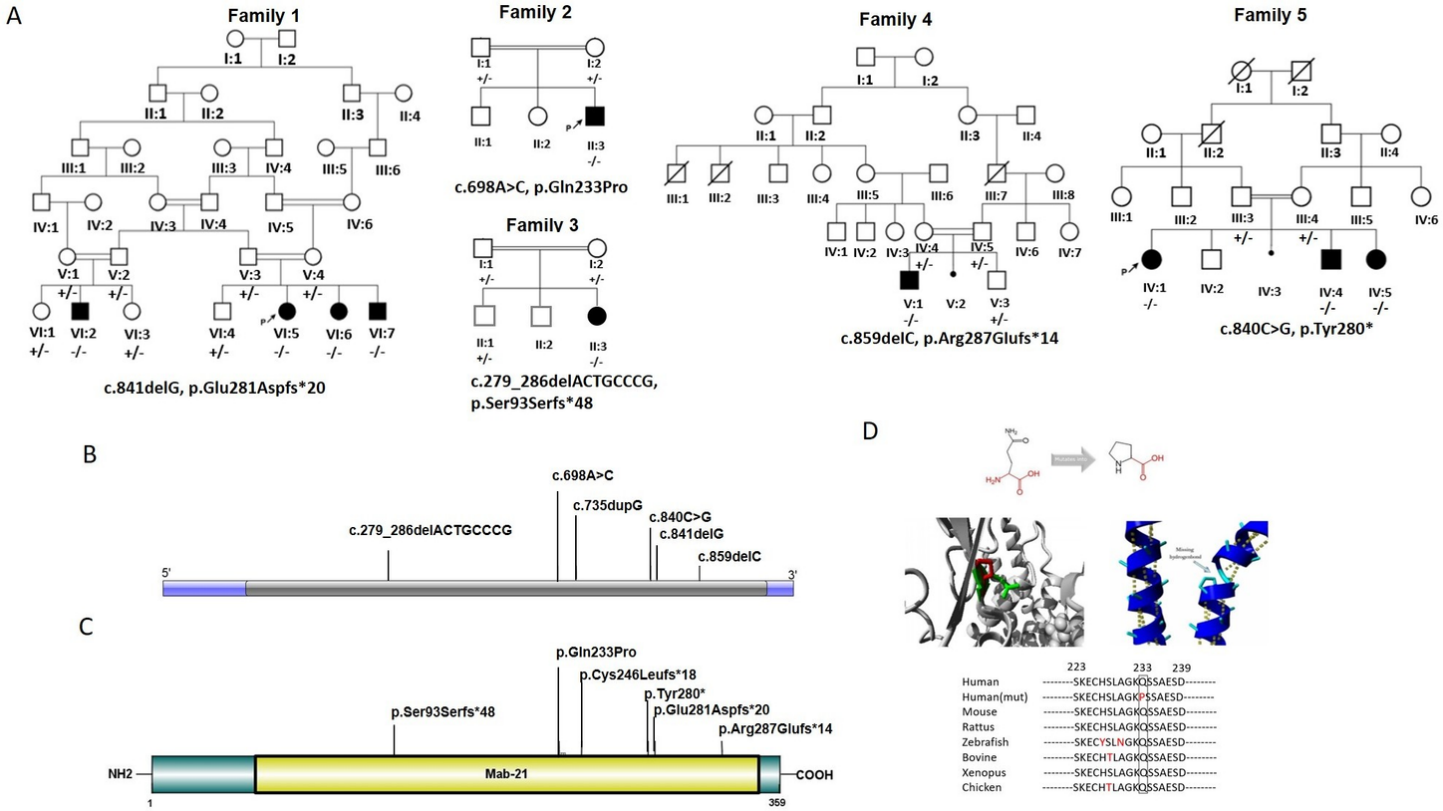
Pedigrees, MAB21L1 mutation segregation patterns and mutation localisation on cDNA and protein level. (A) Pedigrees of four families harbouring biallelic MAB21L1 loss-of function variants identified by ES. In total, nine affected individuals were identified. Wildtype alleles are indicated by ‘+’, alleles carrying identified variants with ‘ -’. (B) Visualisation of the *MAB21L1* gene and COFG associated alleles identified by us as well as the previously identified MAB21L1 allele Bruel *et al*.^12^ (C) Schematic overview of localisations of COFG alleles on protein level. (D) Computational structural model of human MAB21L1, Visualisation of the aminoacid change identified in family 2 and predicted effect on protein folding/structure and evolutionary conservation of the mutated position among species. The variant is predicted to disrupt hydrogen bond formation within the alpha-helix structure, disrupting the protein secondary structure. COFG, Cerebello-Oculo-Facio-Genital; ES, exomic sequencing.

Family 1 was a large consanguineous family from Iran with four affected individuals from two branches, two females and two males, aged between 7 months and 17 years. All affected individuals were born to healthy consanguineous parents following uneventful pregnancies (figure 2). The affected children presented with global developmental delay (DD) and/or moderate-to-severe ID with speech impairment and behavioural abnormalities. Achievement of neurodevelopmental milestones was delayed in all affected ones: walking could not be achieved until the age of 4 years and 2.5 years. All had poor balance with wide-based gait, although this improved considerably with age in individual VI:2. The affected sisters spoke only a few words and displayed an aggressive behaviour and were diagnosed with attention deficit and hyperactivity disorder. Their affected cousin had slurred speech with delayed language acquisition around 5–6 years of age. His behaviour was characterised as initially aggressive but then he developed a shy demeanour. All presented with similar craniofacial dysmorphism with coarse facies, medially sparse/flared and laterally extending eyebrows, synophrys, buphthalmos, anteverted nares, a long and tented philtrum, flat nasal bridge, low anterior hairline and hirsutism. The ophthalmological examinations revealed horizontal nystagmus, strabismus, dry eye and bilateral corneal dystrophy (figure 2) with poor vision necessitating multiple surgeries. Although head circumference measurements were at the lower end of the reference normal range, height and weight were within normal range for age (table 1). Neuroimaging in two affected ones revealed cerebello-vermian hypoplasia and mild Dandy-Walker malformation (figure 1H–M).

The affected males (VI:2 and VI:7) had bilateral agenesis of scrotum with normal median raphe, flat and non-rugose perineal skin (figure 1). Individual VI:7 had undescended testes of normal size and underwent surgery for neoscrotum reconstruction and left orchidopexy (figure 1E). Similarly, the two affected sisters (VI:5 and VI:6) showed absence of labia majora and small labia minora (figure 2F). Individual VI:2 displayed a slightly muscular build with prominent trapezius muscles, markedly underdeveloped and widely spaced nipples with no visible areolae and a tuft of terminal hair extruding from the lactiferous ducts (figure 1G). Skeletal, renal, cardiac, dermal or gastrointestinal anomalies were not observed. There was no history of seizures, and hearing was normal. Karyotype analysis by G-banding and SNP array genotyping did not reveal any pathogenic copy number variants. A clinical suspicion of mucopolysaccharidosis was ruled out by screening for metabolic lysosomal storage disorders in blood and urine.

Family 2 was an Iranian family with one affected male and two healthy siblings, born to healthy first-cousin parents (figure 2). The male index case was born at 38 weeks of gestation via elective caesarean section, following a pregnancy complicated by late gestational diabetes. The mother had opted for Noninvasive Prenatal Testing due to advanced maternal age of 42, which resulted as low-risk for trisomies 13, 18, 21 and sex chromosome aneuploidies. At birth, he had a weight of 3.6 kg (+0.08 SD), length of 56 cm (+2.23 SDs) and head circumference of 34 cm (−0.54 SD). Physical examination revealed a low anterior hairline with flared eyebrows, complete scrotal agenesis and glanular hypospadias with testes palpable under perineal skin. Karyotyping showed normal male constitution of 46, XY. Genitourinary and perineal superficial ultrasound did not reveal any additional malformations. Serum testosterone, 17-OH progesterone and cortisol levels were within normal range. Limited visual fixation and nystagmus were noted during visits to genetics, ophthalmology and neurology clinics. Lack of visual fixation, inability to follow a target and a variable, high-angle esotropia were noted during an ophthalmology examination at 13 months of age. Corneal examination was remarkable for bilateral, subepithelial haze. Fundoscopy showed bilateral pigment granularity consistent with early retinal degeneration and bilateral, mild optic atrophy. Brain MRI showed generalised cerebellar hypoplasia including the vermis. The optic nerves had a mildly thin calibre and a tortuous, redundant appearance. The optic chiasm, pituitary stalk, posterior pituitary lobe and anterior pituitary lobe were unremarkable (figure 1). He started to walk at 18 months of age, was able to climb stairs up and down with assistance and could speak 5–6 words at 26 months of age. Growth parameters were normal (table 1).

Family 3 is a Lebanese Shia family with a single affected girl, third-born to first-degree cousin parents. The affected’s elder sibling was reported to have hydrocephalus and global DD. She was born at 38 weeks of gestation, with normal birth parameters. At 4 months of age, she was referred to the paediatric clinics due to hypotonia, DD and dysmorphic features, comprising a short forehead, medially flared, thick eyebrows with long eyelashes, strabismus and wide nasal tip with short columella. She had bilateral corneal opacities and absence of the labioscrotal folds with non-rugose perineal skin and mild clitoral hypertrophy. Cranial MRI showed severe cerebellar and vermian hypoplasia with a large parietal cyst. A work-up including routine biochemical tests, thyroid function tests, metabolic screening, karyotype analysis, echocardiogram and abdominopelvic ultrasound, revealed normal results. She had severe gastro-oesophageal reflux disease in infancy, which ameliorated with time. Height, weight and head circumference at follow-up at 2 years of age was 116 cm (−1.70 SD), 20 kg (−1.18 SD) and 50 cm (−1.50 SD). She was unable to sit unsupported, walk, babble or speak words.

Families 4 and 5 represent consanguineous Turkish families previously described in detail^13^: family 4 presented with one affected male and family 5 comprised three affected siblings (figure 2). All affected ones presented with a previously unreported phenotype leading to the clinical delineation of the syndrome, comprising agenesis of labioscrotal folds, distinct facial features, nystagmus, corneal dystrophy, low frontal hairline, hairy nipples, cerebellar hypoplasia with pontine involvement and global DD and/or ID (figure 1 and table 1). Patient 7 from family 4 underwent penetrating keratoplasty and extracapsular cataract extraction at the ages of 7 and 18 months, respectively. He was then followed up regularly, but no further operations were performed, and he was functionally blind when examined at the age of 18 years. Patients 7 and 8 from Family 4 were also followed up due to anterior corneal dystrophy with opacities, but visual loss was limited, necessitating no operations.

ES was performed on single affected individuals from families 1 and 5, the affected and an unaffected sister for family 4 and as Trio Exomein families 2 and 3. Assuming the disease follows an autosomal recessive inheritance in these families due to presence of consanguinity and multiple affected siblings, we prioritised potentially functional homozygous variants residing within runs of homozygosity larger than 1 Mb. These variants were screened through publicly available population databases (gnomAD, GME, Iranome) and in-house database generated for genetic variant frequency in human population. We excluded synonymous variants, intronic variants (>5 bp from exon boundaries) and variants with >1% minor allele frequency. Homozygous variants were prioritised in consanguineous families; however; compound heterozygous variants were not primarily filtered out. In families 2 and 3, we further excluded variants also found in the exome of a parent in homozygous state and in family 4, all variants the unaffected sister was homozygous for were likewise excluded.

ES on individual 1_VI:5 identified a novel homozygous truncating variant in *MAB21L1* (NM_005584.4, c.841delG, p.Glu281Aspfs*20) located within a region of homozygosity (chr13: 37,227,227–37,247,380, online supplementary figure S1), segregating with the disease phenotype in the family. Homozygous variants in other genes detected by ES (online supplementary table S1) were excluded by Sanger sequencing segregation analysis. Parallel Trio -ES analysis in family 2 revealed a novel homozygous missense variant (NM_005584.4, c.698 A>C, p.Gln233Pro) in *MAB21L1,* located within a long stretch of homozygosity (chr.13: 24 737 091–43 447 499, online supplementary figure S1). This position is conserved across species^21^ (figure 2) and the variant is predicted to have damaging effects by SIFT, MutationTaster and CADD, while PolyPhen, Fathmm and PROVEAN predict the change to be benign. Modelling using HOPE (http://www.cmbi.ru.nl/hope/method/) revealed that the mutated residue locates to an alpha-helix and that size difference and difference in hydrophobicity is predicted to affect formation of hydrogen bonds at the mutated position. Further, proline disrupts alpha helix formation if not located within the first 3 residues of the structure. The variant is therefore predicted to disrupt the alpha helix which in turn is likely to affect the secondary protein structure (HOPE prediction, figure 2).

10.1136/jmedgenet-2018-105623.supp1 Supplementary data

In family 3, a novel homozygous frameshift mutation in *MAB21L1* (NM_005584.4, c.279_286delACTGCCCG, p.Ser93Serfs*48) was identified in individual of the affected child by trio ES analysis.

In family 4, individual V:2 and the healthy sister were investigated by Whole Exome Sequencing (WES) which uncovered a novel homozygous truncating *MAB21L1* variant (NM_005584.4, c.859delC, p.Arg287Glufs*14) while in individual IV:1 from family 5 WES revealed a novel homozygous stop-gain variant in *MAB21L1* (c.840C>G; p.Tyr280*).

All identified variants were validated and cosegregation analysis was performed by Sanger sequencing (see online supplementary figure S2). No other likely pathogenic variants compatible with the phenotype were identified in currently known disease-causing genes in ES data. Variants left after filtering are shown in online supplementary table S1 for family 1.

As the function of MAB21L1 has remained rather elusive, we proceeded to perform an analysis of putative MAB21L1 protein interactions via crosstalk visualisation using the String Network.^20^ This revealed connections between MAB21L1 and HOX, MEIS1-2 and PBX1-3 genes as well as LMX1B^20^ in addition to its function downstream of Pax6 and upstream of FOXE3 (online supplementary figure S3). Mutation in genes of those developmental pathways cause a range of other developmental disorders in mammals (online supplementary table S2), providing possible pathomechanistic explanations for the phenotype observed as a consequence of MAB21L1 loss of function in humans.

## Discussion

Congenital malformations of the labioscrotal folds are very rare with complete agenesis of the scrotum being one of the rarest. Over the past 30 years, there have been only a handful of documented reports of patients who had congenital agenesis of the scrotum without an otherwise identifiable genetic syndrome. Bruel *et al* very recently described a homozygous loss of function mutation in *MAB21L1* in such a case^12^ Here, we identify homozygous variants in *MAB21L1* in 10 affected members of 5 consanguineous families who present with congenital underdevelopment of the labioscrotal folds in a similar overlapping clinical phenotype. The disease follows an autosomal recessive mode of inheritance in all families. Moderate-to-severe DD/ID, behavioural abnormalities, severe cerebellar hypoplasia, a noticeably short forehead, medially sparse/flared and laterally extending eyebrows, corneal dystrophy, underdeveloped labioscrotal folds and tufts of hair extruding from the lactiferous ducts with breast and nipple underdevelopment are the main features. Additional features such as pontine involvement, retinal degeneration, anteverted nares and low set ears were variably observed in the affected individuals (table 1 and figure 2).

The mutations in *MAB21L1* identified in this study as well as the previously identified variant in an additional family with a similar phenotype are frameshift/nonsense variants except one missense allele, most likely leading to complete loss of function of the protein, including loss of interactions with potential partner proteins. In mice, highest *Mab21* expression levels have been detected in the rhombencephalon, cerebellum, midbrain and prospective neural retina.^9^ The precise function of *MAB21L1* during embryonic development and how *MAB21L1* mutations cause COFG syndrome has not been established yet. However, *Mab21l1* deficient mice and *C. elegans* show a phenotype relating to the human disease pattern with abnormalities noted in the brain, eyes, movement and reproductive organs. Specifically, abnormalities observed in loss-of function mouse models including impaired notochord and neural tube differentiation, rudimentary lens, absence of iris and ciliary cells, preputial glands and calvarial osteogenesis^4^ show a pattern resembling the human COFG phenotype.

On the basis of conserved sequences of members of the MAB21 protein family sharing over 90% aminoacid similarity, the family seems related to the larger family of nucleotidyltransferases (NTases) and *MAB21L1* shares considerable sequence homology with the cyclic GMP-AMP synthase.^6^ However, Oliveira Mann *et al* suggested MAB21 has moderate binding to ssRNA, but they did not refute the NTase activity. In vitro, *MAB21L1* seems to be able to oligimerise,^6^ to which extent this phenomena may also play a role in vivo has not been investigated to date. MAB21L1/2 have a similar tissue expression pattern, bringing up the question to which extend they may be functionally redundant. For human, our data argue against redundancy and suggest essential individual functions during development at least with relation to the eyes, the central nervous system (CNS), parts of the craniofacial and the genital tissues.

In *C. elegans*, it has been reported that *Mab21* is negatively regulated by *cet-1* (vertebrate BMP4)^5^ and likewise in Xenopus *MAB21* suppresses BMP4, suggesting a role within or downstream of the TGF-ẞ pathway. Likewise, murine embryonic development and organogenesis indicate crucial involvement of Mab21l1 during eye development with high expression levels in both the optic vesicle as well as the lens placode, where its seems to act downstream of Bmp/FGF regulated PAX6 but upstream of Foxe3.^4^ In line with this, our String Network analysis (online supplementary figure S3)^20^ suggests that that *MAB21L1* is connected to *HOX*, *MEIS1-2* and *PBX1-3* genes as well as *LMX1B* (online supplementary figure S3). Human mutations in these putative interaction partners have been found to cause specific genetic syndromes including Myhre syndrome (OMIM#139210, *SMAD4* variants), Hand-foot-genital syndrome (OMIM#140000, *HOXA13* mutations) or Nail-patella syndrome (OMIM#161200, *LMX1B* dysfunction) and congenital malformation involving the skeleton, eye, genitals, kidney, heart and brain (*BMP2, PAX6, MEIS2*). Interestingly, these mutations have been found to be inherited in a predominant autosomal dominant fashion in contrast to the recessive variants we identified in *MAB21L1* (online supplementary table 2). Regarding the potential role Mab21l1 may play for proper skeletal development, Kim *et al* further showed that activation of the JNK1/MKK4 pathway results in MEF phosphorylation and subsequent Mab21l1 activation in osteoblast cells.^22^

In summary, in this study, we confirm that biallelic *MAB21L1* loss-of-function mutations cause an extremely rare autosomal recessive recognisable syndrome, COFG syndrome. Likely, the developmental defects observed result at least partially from impaired TGF-beta/BMP and JNK1/MKK4 signalling; however; further functional studies regarding the characteristics observed in this syndrome will be required to determine the precise downstream cell signalling defects resulting from human *MAB21L1* loss of function variants.
