# Supplementary material for: MAB21L1 loss of function causes a syndromic neurodevelopmental disorder with distinctive cerebellar, ocular, craniofacial and genital features (COFG syndrome)
Source: J Med Genet. 2018 Nov 28;56(5):332–9. doi: 10.1136/jmedgenet-2018-105623 (PMC6581149; doi:10.1136/jmedgenet-2018-105623)
Supplement: Supplementary data [file jmedgenet-2018-105623supp001.docx]

| **Family** | **Variant** | **Gene** | **AA change** | **dbSNP** | **Allele frequency in Gnomad** | **OMIM No.** |
| --- | --- | --- | --- | --- | --- | --- |
| 1 | chr2:97164934 G>C | NEURL3 | NM_001285486:exon5:c.C190G, p.P64A | - | - | 617206 |
| 1 | Chr2:109411047 G>A | CCDC138 | NM_144978:exon5:c.G446A, p.S149N | - | - | - |
| 1 | chr10:128810594 T>C | DOCK10 | NM_001380:exon12:c.T1094C, p.I365T | - | - | 611518 |
| 1 | chr1:89225946 T>G | PKN2 | NM_006256:exon3:c.T391G, p.C131G | rs755666862 | 6.271e-5 | 602549 |
| 1 | chr1:120284468 C>T | PHGDH | NM_006623:exon10:c.C1157T, p.A386V | rs768396645 | 1.804e-5 | 606879 |
| 1 | chr2:128396897 G>A | LIMS2 | NM_001256542:exon7:c.C529T, p.R177C | rs528266998 | 2.438e-5 | 607908 |
| 1 | chr13:110437908 C>T | IRS2 | NM_003749:exon1:c.G493A, p.A165T | - | - | 600797 |
| 1 | chr22:42076296 C>T | NHP2L1 | NM_005008:exon3:c.G76A, p.V26I | - | - | 601304 |
| 3 | Chr2: 54856890 C>A | SPTBN1 | NM_003128.2: c.2619C>A, p.Asn873Lys | - | - | 182790 |
| 3 | Chr10: 76780901 G>A | KAT6B | NM_001256468.1: c.2330G>A, Arg777Gln | - | 0,0000082 | 605880 |
| 3 | Chr13: 24465620 T>C | C1QTNF9B | XM_005266372.1: c.296A>G, p.Glu99Gly | - | 0,0000083 | 614148 |
| 3 | ChrX: 56591294 G>T | UBQLN2 | NM_013444.3: c.988G>T, p.Ala330Ser | - | 0,0000056 | 300264 |

**Table S1:** Homozygous rare variants identified after filtering whole exome sequencing data of the index patients from family 1.

| Gene | Disease and related phenotype | | Mode of Inheritance | OMIM No. |
| --- | --- | --- | --- | --- |
| *MAB21L2* | Microphthalmia/coloboma and skeletal dysplasia syndrome | | AR, AD | 604357 |
| *SMAD4* | Myhre syndrome | | AD | 139210 |
| *BMP2* | Craniofacial, Skeletal, and  Cardiac Features | | AD | ---- |
| *PAX6* | Aniridia, Cataract with late-onset corneal dystrophy | | AD | 607108 |
| *HOXA13* | hand-foot-genital syndrome | | AD | 140000 |
| *LMX1B* | Nail-patella syndrome | | AD | 161200 |
| *MEIS2* | Cleft palate, cardiac defects, and mental retardation | | AD | 600987 |
| *PBX1* | Congenital anomalies of kidney and urinary tract syndrome with or without hearing loss, abnormal ears, or developmental delay | | AD | 617641 |
| *RUNX2* | Cleidocranial dysplasia, forme fruste, with brachydactyly | | AD | 119600 |
|  |  |  | |  |

**Table S2.** Identified human mutations in interaction- partners of MAB21L1 and resulting human phenotypes

**A**

**Chr.13: 37,227,227- 37,247,380**


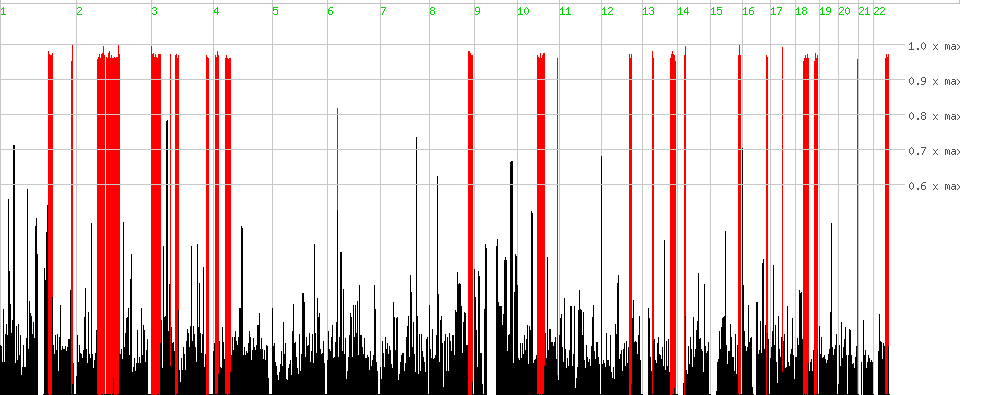


Figure S1.

**B**

*1*. chr1: 65,368,561-88,605,099 
*2*. chr.4: 37,156,452-63,243,416
*3.* chr.6: 184,718-11,236,644
*4*. chr.7: 29,198,773-83,173,734 
*5*. chr.9: 30,064,498-40,087,758 
*6*. chr.9: 71,013,799-82,975,578
7. chr.11: 8,017,223-22,696,112
8. chr.11: 46,556,751-51,563,636
9. **chr.13: 24,737,091-43,447,499**10. chr. 17: 18,900-6,027,060

**Figure S1.** **Homozygous areas identified by SNP chip analysis.** (A) Homozygous intervals identified in family 1 (homozygosity mapper). The MAB21L1 variant lies within a homozygous interval on chr13: 37,227,227- 37,247,380. (B) Homozygous intervals identified in family 2 (homozygpsity mapper). The MAB21L1 varaint lies within a homozygous interval in chromosome 13: 24,737,091-43,447,499 .


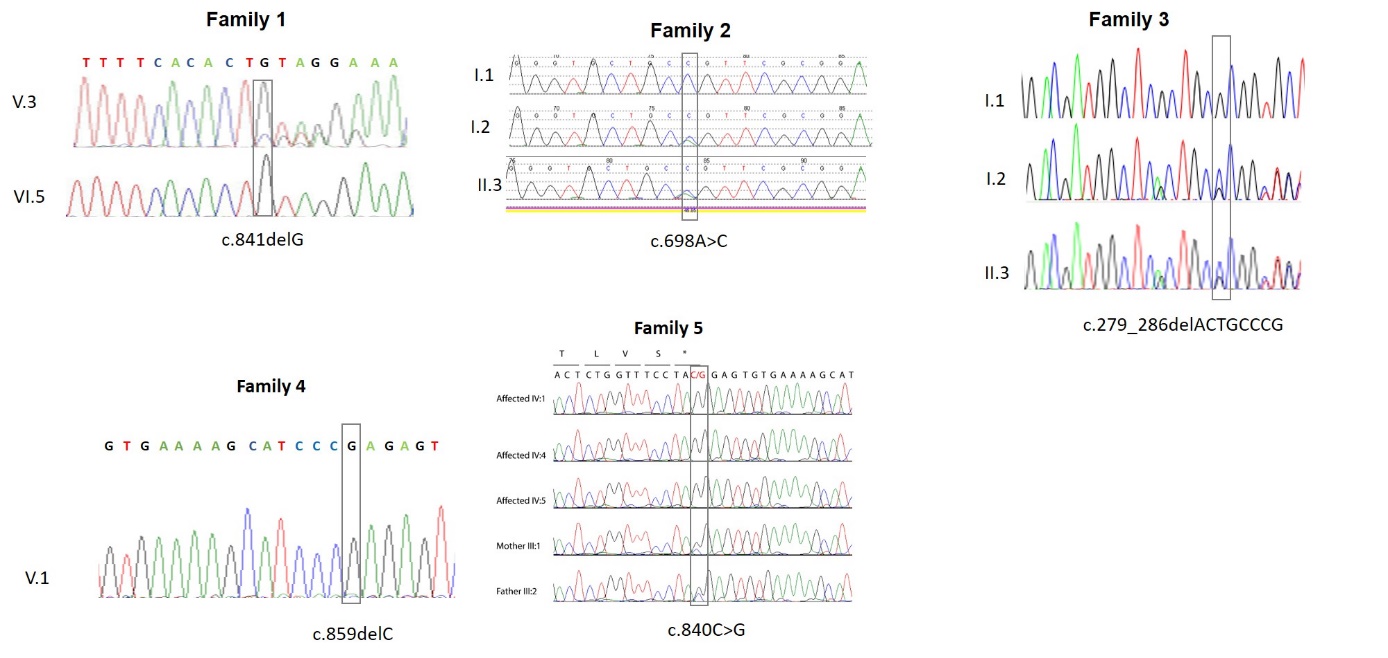


**Figure S2: Sanger sequencing chromatograms of identified MAB21L1 disease causing alleles.**

**A**


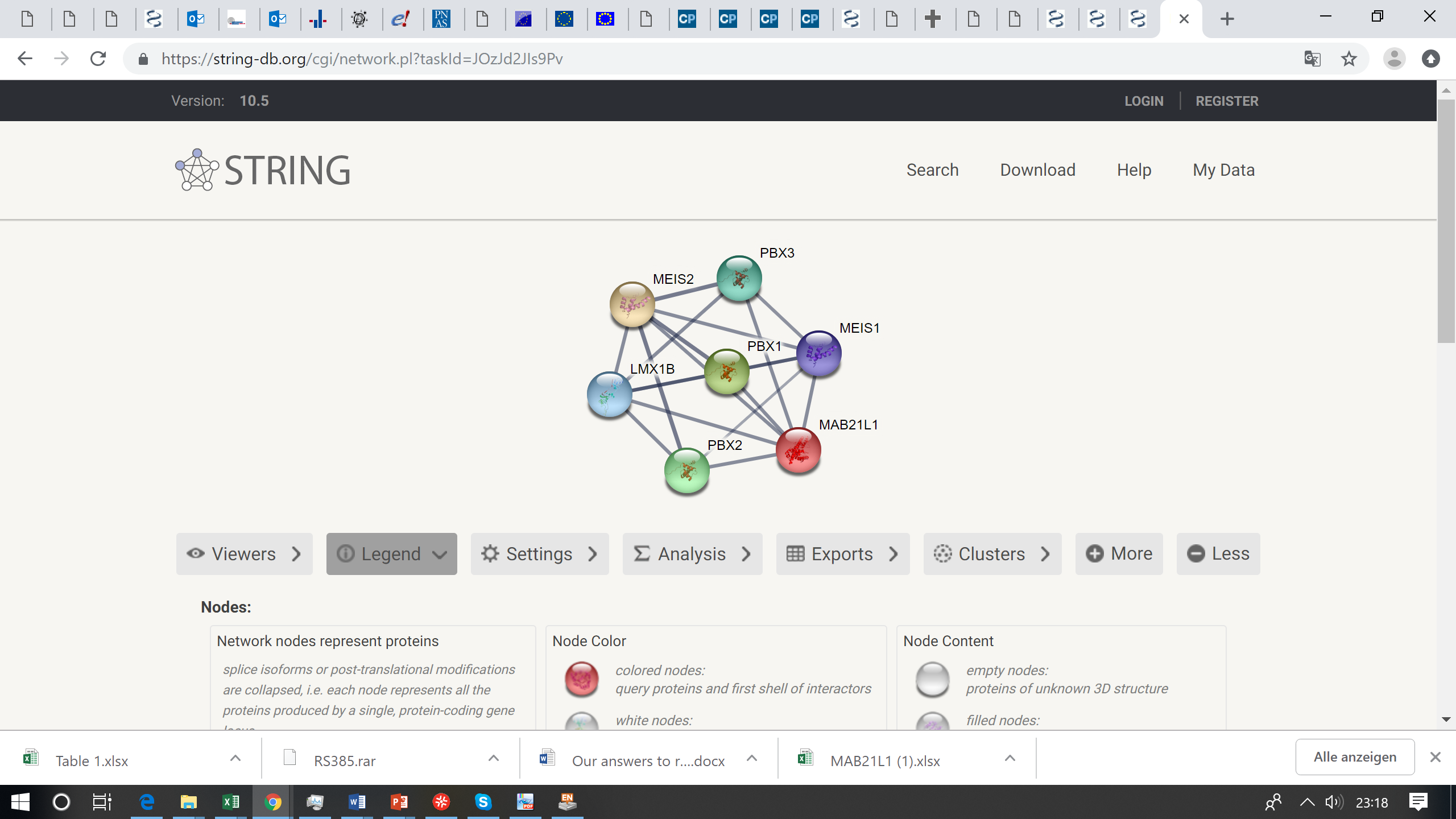


**B**


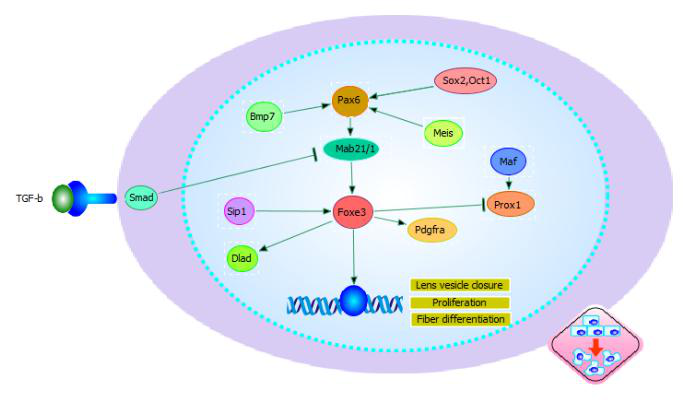


**Figure S3: Crosstalk visualization for MAB21L1 and related proteins. (A)** MAB21L1 experimentally confirmed protein-protein interactions (String network analysis). Analysis setup was chosen so that only experimentally confirmed interactions partners are displayed. Confidence levels were High (>0.70) for all MAB21L1 interaction proteins displayed. **(B)** Schematic of MAB21L1 putative downstream effects regulating ocular (lens) development
